# *Macrotyloma axillare* ‘Java’ presents structural and biochemical responses against *Meloidogyne javanica*

**DOI:** 10.3389/fpls.2025.1599195

**Published:** 2025-07-24

**Authors:** Angélica Miamoto, João Paulo Rodrigues Marques, Cláudia Regina Dias-Arieira

**Affiliations:** ^1^ Graduate Programme in Agronomy, State University of Maringá, Maringá, PR, Brazil; ^2^ Department of Basic Science, Faculty of Animal Science and Food Engineering, University of São Paulo, Piracicaba, SP, Brazil

**Keywords:** root-knot nematode, giant cells, histopathology, preexisting defenses, Inducible defenses, plant defense

## Abstract

The genus *Meloidogyne* is one of the most significant groups of plant-parasitic nematodes. Plant species capable of inhibiting the development and reproduction of this pathogen can be utilized as a management strategy. This study aimed to analyze the structural (constitutive and induced) and biochemical defense responses of the legume *Macrotyloma axillare* 'Java' in interaction with *Meloidogyne javanica*. The response of 'Java' to *M. javanica* was evaluated in two trials by inoculating 2000 eggs and second-stage juveniles (J2), with tomato used as a susceptible control. The reproduction factor (RF) was assessed 60 days after inoculation (DAI). Histochemical tests were conducted to observe constitutive and induced defense traits in 'Java' and tomato at 10, 20, and 30 DAI. Additionally, the reaction with 3,3'-diaminobenzidine was evaluated at 5 DAI, and root tips were examined using scanning electron microscopy at 30 DAI. The total protein content in roots was also measured at 8 and 12 DAI. In both trials, 'Java' showed RF < 1 (0.7 and 0.8) and was considered resistant to *M. javanica*, while tomato was susceptible, with RF > 1 (16.7 and 21.2). Histochemical analyses revealed constitutive defenses in 'Java', including the accumulation of phenolic compounds in phloem ducts and widespread suberin thickening in endodermal cells, whereas tomato exhibited only limited suberin thickening. Induced defenses in 'Java' included nematode females surrounded by cells with phenolic compound and protein accumulation, as well as deformed females and malformed giant cells with few cell wall invaginations at feeding sites. In tomato, feeding site development and nematode reproduction occurred without the accumulation of defensive compounds. Reaction with 3,3'-diaminobenzidine was more pronounced in 'Java' (42%) compared to tomato (23%). Scanning electron microscopy showed that giant cells in 'Java' were, on average, 46% smaller than those in tomato. Furthermore, 'Java' exhibited higher total protein levels when inoculated with *M. javanica* compared to the non-inoculated plant or tomato. In conclusion, 'Java' exhibits both constitutive and induced defense traits, which inhibit the full development and reproduction of *M. javanica* in its roots.

## 1 Introduction

The genus *Meloidogyne* (root-knot nematode) is one of the major groups of plant-parasitic nematodes, capable of causing significant damage to various crops. As sedentary endoparasites, these nematodes establish complex interactions with their hosts by invading the roots and secreting substances from their esophageal glands. This process induces cellular hypertrophy and hyperplasia, resulting in the formation of a feeding site known as giant cells (Bozbuga et al., 2018). These cells act as metabolic sinks, diverting the plant’s nutrients toward the nematodes, thereby impairing plant development to the pathogen’s benefit (Perry et al., 2009).

In this context, plants capable of inhibiting the full development of the feeding site can reduce nematode reproduction. A plant’s ability to interfere with a pathogen’s parasitism is associated with its defense mechanisms, which may be biochemical, molecular, or morphological, and can be either preexisting or induced following infection (Qidwai et al., 2021).

Preexisting or constitutive defense mechanisms include the plant’s natural physical barriers, the most common being the cell wall, cuticle, lignin, cellulose, pectin, and suberin, which can protect the plant from pathogen invasion (van Baarlen et al., 2007). Biochemical barriers, on the other hand, consist of secondary metabolites produced by the plant, such as phenols, proteins, alkaloids, terpenes, and others. These compounds can block or even kill pathogens during invasion attempts and may interact with structural barriers, enhancing the plant’s resistance (Doughari, 2015).

If the pathogen overcomes preexisting barriers and penetrates host tissues, the plant can still express induced defense mechanisms, which are often more prominent than preexisting resistance. Induced resistance can be structural, leading to increased epidermal wall thickness, waxy cuticle deposition, and enhanced cell wall size and rigidity (Doughari, 2015). It can also be biochemical, involving the production of compounds with detrimental effects on the pathogen. In such cases, parasitism triggers various chemical and molecular signaling cascades, activating plant resistance genes that recognize pathogen-associated molecular patterns (PAMPs) and express resistance through the production of proteins, enzymes, lipids, carbohydrates, phenols, and other cell wall derivatives (Dodds and Rathjen, 2010).

Structural and biochemical traits in response to pathogenesis are essential factors to investigate in plant-pathogen systems. Consequently, there is growing interest in identifying plants with both constitutive and induced defense mechanisms against plant-parasitic nematodes, as such plants could be incorporated into integrated management systems. One plant that shows great potential in this regard is *Macrotyloma axillare* ‘Java.’ This species exhibits resistance to *Meloidogyne javanica*, delaying nematode development after penetration, inhibiting the full formation of feeding sites, and causing deformation of adult females (Miamoto et al., 2020). It is also known that *M. axillare* ‘Java’ expresses high levels of peroxidase in the presence of the nematode (Miamoto et al., 2022a). This enzyme reduces hydrogen peroxide levels, rendering the cellular environment toxic and unfavorable for pathogens by regulating the production of reactive oxygen species (ROS) (Passardi et al., 2005; Schaffer and Bronnikova, 2012).

However, the mode of action of *M. axillare* ‘Java,’ whether constitutive or induced, has not yet been fully elucidated. Therefore, further studies are required to clarify this aspect. Thus, the objective of this study was to analyze the structural and biochemical responses of the interaction between *M. axillare* ‘Java’ and *M. javanica*.

## 2 Materials and methods

### 2.1 General characteristics of the experiments

The experiments were conducted partly in a greenhouse and partly in a laboratory.

The *M. javanica* inoculum used in the experiments was obtained from a pure population maintained on tomato (*Solanum lycopersicum* L.) ‘Santa Clara’ for 60 days. The eggs and second-stage juveniles (J2) were extracted using the method adapted from Boneti and Ferraz (1981) and counted in a Peters chamber under a light microscope at 40x magnification.

### 2.2 Reproduction factor of *M. axillare* ‘Java’ to *M. javanica*

The experiments were conducted from July to September 2021 (Trial 1) and from September to November 2021 (Trial 2). The experimental design was completely randomized, with two treatments: *M. axillare* ‘Java’ and *S. lycopersicum* ‘Santa Clara’ (susceptible control), and six replicates.

For this purpose, *M. axillare* ‘Java’ and *S. lycopersicum* plants were grown in polystyrene trays containing commercial substrate (Bioplant Agrícola Ltda.) and transplanted 20 days after germination into 500 mL polystyrene pots filled with a 1:1 sterilized mixture of soil and sand, autoclaved for 2 hours at 120°C.

Two days after transplantation, the plants were inoculated with 2,000 eggs and second-stage juveniles (J2) of *M. javanica*, using a 2 mL suspension, which were obtained by the method proposed by Boneti and Ferraz (1981). The plants were cultivated for 60 days in a greenhouse. After this period, the roots were separated from the shoots, washed, and weighed using a semi-analytical balance. The nematodes were then extracted and counted following the methodology previously described. The total number of nematodes and the reproduction factor (RF = final population/initial population) were subsequently determined (Oostenbrink, 1966).

### 2.3 Histochemical tests

For the histochemical tests, four seeds of *M. axillare* ‘Java’ and *S. lycopersicum* ‘Santa Clara’ were sown in 180 mL pots containing a sterilized soil:sand mixture (1:1), autoclaved for 2 hours at 120°C. After germination, thinning was performed, leaving only one plant per pot. The plants were then inoculated with 2000 eggs and second-stage juveniles (J2) of *M. javanica*. Cultivation was carried out between August and September 2021.

Four roots per plant were carefully removed and washed for evaluations at 10, 20, and 30 days after inoculation (DAI). Root preparation for sectioning followed the methodology described by Pegard et al. (2005). Root fragments were fixed for 24 hours in a solution containing 2% paraformaldehyde and 1% glutaraldehyde in 0.1 M phosphate buffer (pH 7.0). The samples were dehydrated in a graded ethanol series (10%, 30%, 50%, 70%, 80%, 90%, and 100%) and embedded in Technovit^®^ 7100 resin following the manufacturer’s protocol. After resin embedding, the blocks were sectioned into 5 μm fragments using a Leica RM 2045 rotary microtome. Sections were placed in drops of distilled water on microscope slides and fixed on a laboratory hot plate. The samples were analyzed under an epifluorescence microscope using A4 and GFP filters. Merged images were captured for further analysis.

For histological characterization, the following stains were used: Toluidine Blue (C_15_H_16_ClN_3_S) for general histological analysis; ferric chloride (FeCl_3_) for phenolic compounds detection; zinc iodide chloride (ZnCl_2_) for starch detection; Ruthenium Red (Cl_6_H_42_N_14_O_2_Ru_3_) and Sudan IV (C_24_H_20_N_4_O) for pectic compounds detection; and Ponceau Xylidine (C_20_H_13_N_2_NaO_4_S) for protein detection (Marques and Soares, 2022). Images were captured using a Leica DC 300F video camera attached to a Leica DMLD microscope, and descriptive sample analysis was performed.

### 2.4 Reactive oxygen species

To analyze reactive oxygen species (ROS), *M. axillare* ‘Java’ plants were cultivated in polystyrene trays containing a commercial substrate (Bioplant Agrícola Ltda.). Twenty days after germination, the plants were carefully removed from the trays, washed, and transferred to 50 mL plastic tubes containing a 30 mL suspension with 500 second-stage juveniles (J2) of *M. javanica*, extracted following the Baermann funnel method (Baermann, 1917), or to tubes containing only water. After five days, the samples were subjected to hydrogen peroxide (H_2_O_2_) detection using the organic compound 3,3’-diaminobenzidine (DAB), which is oxidized by hydrogen peroxide, resulting in a dark brown stain in the root system (Giordani et al., 2021; Marques and Soares, 2022).

The samples were photographed using a three-dimensional digital microscope (Hirox KH-8700, JP). Analysis of 100 root segments of *M. axillare* ‘Java’ or *S. lycopersicum* was performed, identifying the presence or absence of ROS.

### 2.5 Scanning electron microscopy

*Solanum lycopersicum* and *M. axillare* ‘Java’ plants were inoculated with 2000 eggs and second-stage juveniles (J2) of *M. javanica*. Thirty days after inoculation, root apices were collected for analysis. The collected materials were subjected to the cryofracture technique described by Marques and Soares (2022).

In this procedure, fragments from inoculated plants were fixed in Karnovsky’s solution for 48 hours, followed by immersion in a cryoprotectant solution (30% glycerin in distilled water) for 2 hours. The samples were subsequently immersed in liquid nitrogen and cryo-fractured with the aid of a scalpel before being immersed in distilled water.

The samples were then dehydrated in an ascending series of acetone concentrations and subjected to critical point drying using CO_2_. The dehydrated samples were mounted on aluminum stubs with carbon adhesive tape and sputter-coated with gold for 200 seconds. Finally, the samples were analyzed using a JEOL scanning electron microscope.

### 2.6 Quantification of total proteins

The experiment was conducted in October 2020, using a completely randomized design with four treatments: *M. axillare* ‘Java’ and *S. lycopersicum* ‘Santa Clara,’ inoculated or not with *M. javanica*, with four replicates. Each sample was analyzed in duplicate.

Four seeds of *M. axillare* ‘Java’ and *S. lycopersicum* ‘Santa Clara’ were sown in 500 mL polystyrene pots containing a soil:sand (1:1) mixture, autoclaved for 2 hours at 120°C. After germination, thinning was performed carefully, leaving only one plant per pot. Fifteen days after germination, the nematode-treated plants received a 1 mL suspension containing 2000 eggs and J2 of *M. javanica*. Four plants from each treatment were harvested at 8 and 12 days after inoculation (DAI). For each sample, the aerial parts were discarded, and the roots were collected and stored separately in aluminum foil at -80°C until analysis.

The samples were macerated in a mortar with liquid nitrogen, adding 1% polyvinylpyrrolidone (PVP) and 4 mL of 50 mM potassium phosphate buffer, pH 7.0, with 0.1 mM EDTA. After this process, the samples, in the form of extracts, were centrifuged at 14,500 rpm for 30 minutes at 4°C. The supernatant, corresponding to the enzymatic extract, was transferred to 1.5 mL microtubes and stored at -80°C until protein quantification and enzymatic activity determination.

For the quantification of total protein content in the samples, the Bradford assay (Bradford, 1976) was performed, with protein concentration determined using a standard curve of concentrations obtained from bovine serum albumin and expressed in mg mL^-^07B;¹ of sample.

### 2.7 Statistical analysis

For the reproduction factor experiments, total protein quantification, and phenolic compound analysis, the original data were transformed using √x to meet the assumptions of normality, as assessed by the Shapiro-Wilk test. The transformed data were then subjected to analysis of variance (ANOVA) (*p* < 0.05), and means were compared using Tukey’s test. For the EROS test, the chi-square test was used. All statistical analyses were performed using the Sisvar software (Ferreira, 2014). Histochemical data were evaluated descriptively.

## 3 Results

### 3.1 Reaction of *M. axillare* ‘Java’ to *M. javanica*

In both trials, *M. axillare* ‘Java’ exhibited lower reproduction compared to *S. lycopersicum* (**Table 1**). Regarding the reproduction factor (RF), in both trials, *M. axillare* ‘Java’ showed a RF < 1 (Trial 1 = 0.7 and Trial 2 = 0.8), while *S. lycopersicum* exhibited a RF > 1 (Trial 1 = 16.7 and Trial 2 = 21.2) (**Table 1**).

**Table 1:** Total number of nematodes (TNN), nematodes per gram of root (NGR), and reproduction factor (RF) of *Meloidogyne javanica* in roots of *Macrotyloma axillare* ‘Java’ and *Solanum lycopersicum* ‘Santa Clara’, 60 days after inoculation with 2000 eggs + second-stage juveniles (Trial 1 and Trial 2).

Treatments	TNN	NGR	RF	TNN	NGR	RF
	Trial 1			Trial 2		
‘Java’	1412 b	518 b	0.7 b	1612 b	500 b	0.8 b
Tomato	33450 a	5680 a	16.7 a	42487 a	8568 a	21.2 a
CV (%)	28.9	24.5	28.9	33.1	60.7	33.1

Means followed by the same lowercase letter do not differ from each other according to Tukey’s test (*p* < 0.05). CV = coefficient of variation.

### 3.2 Histochemical tests

#### 3.2.1 Constitutive traits

**Figure 1:**
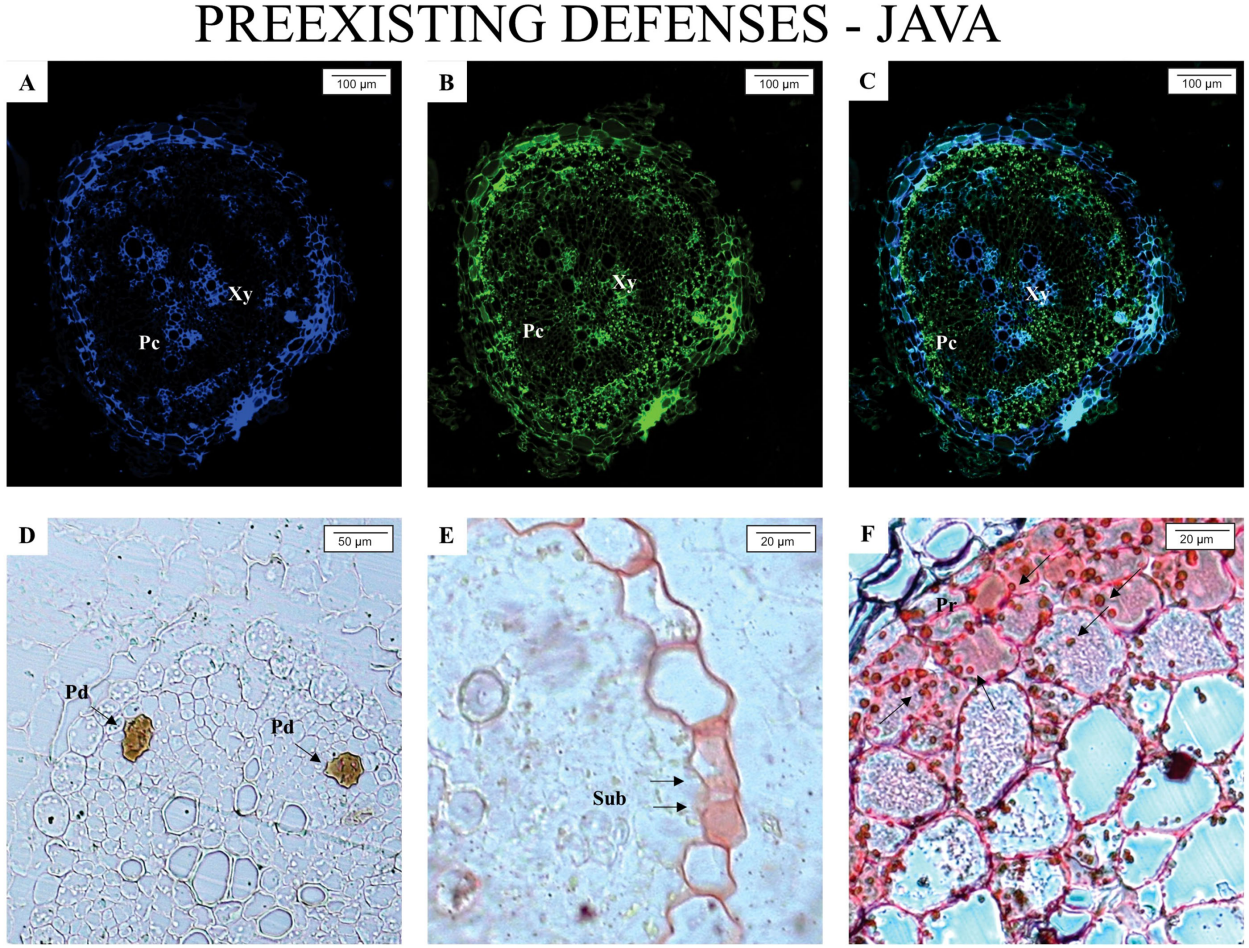
Epifluorescence and light microscope analysis of roots from *Macrotyloma axillare* ‘Java’ not parasitized by the nematode. All images represent the pre-existing defense mechanisms in *M. axillare* ‘Java’. **(A–C)** presence of phenolic compounds (Pc); **(D)** presence of resin ducts (Pd) in the phloem; **(E)** suberin thickening (Sub) in the vascular cylinder; **(F)** protein accumulation (Pr). Xy, xylem.

**Figure 2:**
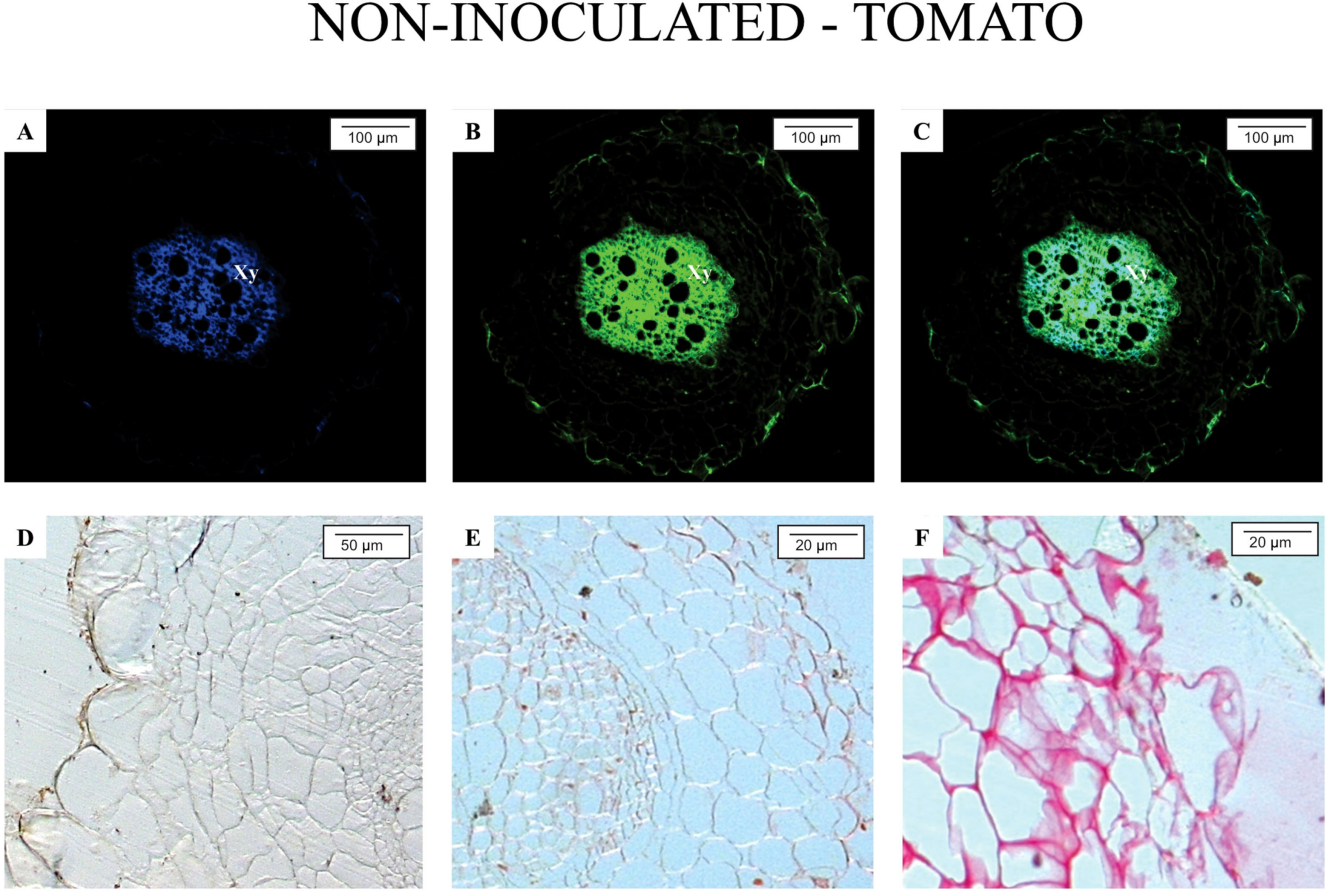
Epifluorescence and light microscopy analysis of *Solanum lycopersicum* ‘Santa Clara’ roots not parasitized by nematodes. Root segments were exposed to the following filters: **(A)** A4; **(B)** GFP; **(C)** merged image of A and B. Histochemical staining revealed: **(D)** absence of phenolic compounds (ferric chloride staining); **(E)** absence of suberin (ruthenium red staining); **(F)** absence of proteins (Ponceau Xylidine staining). *Xy*, xylem.

**Figures 1** and **2** illustrate the anatomical and biochemical characteristics of non-inoculated roots from *M. axillare* ‘Java’ and *S. lycopersicum*, respectively. These images reveal the baseline defensive architecture and autofluorescent profiles that may contribute to differential susceptibility to *M. javanica*.

In *M. axillare* ‘Java’ roots (**Figure 1**), a variety of pre-formed defense features were detected. Under GFP and A4 filters, there is a notable and consistent autofluorescence pattern associated with phenolic compounds (**Figures 1A–C**), strongly suggesting the constitutive presence of these secondary metabolites. These compounds, which fluoresce under specific excitation, are known for their antimicrobial properties and structural reinforcement of plant tissues. In **Figure 1D**, the presence of resin ducts in the phloem was observed, representing an additional anatomical barrier with potential deterrent or toxic effects on invading organisms. Panel E highlights suberin deposition within the vascular cylinder—another chemical defense contributing to reduced permeability and pathogen entry. Lastly, Panel F shows strong fluorescence linked to protein accumulation, possibly including pathogenesis-related proteins or structural proteins that reinforce cell walls.

This complex of constitutive features in *M. axillare* ‘Java’ roots represents a robust pre-invasion defense system. The widespread and intense autofluorescence across multiple tissue types and cellular components confirms the metabolic investment in chemical and physical defense barriers even in the absence of biotic stress. These defenses likely contribute to the resistance observed in Java plants upon nematode challenge, as discussed previously.

In contrast, roots of *S. lycopersicum* (**Figure 2**) revealed a marked absence of such defense characteristics. Autofluorescence was minimal, with only faint background signals, particularly in the vascular tissue, and no clear indication of phenolic accumulation, suberin layers, or specialized structures such as resin ducts. The lack of visible fluorescence under both A4 and GFP filters indicates a low baseline level of defensive metabolites. This biochemical and anatomical simplicity may partially explain the tomato’s high susceptibility to *M. javanica*, as the plant lacks structural and chemical barriers that could prevent initial infection or nematode establishment.

Overall, the comparison between Java and tomato roots underlines the role of constitutive defenses in resistance. While Java exhibits a multi-layered and metabolically active defense system prior to infection, tomato roots remain relatively unprotected, relying likely only on inducible responses after pathogen recognition, which, as shown in previous analyses, are insufficient to prevent nematode development. The use of fluorescence microscopy with specific filters provides valuable insights into the spatial distribution and intensity of these natural defense compounds, offering a non-invasive and efficient approach for screening resistant genotypes.

#### 3.2.2 Induced traits

**Figure 3:**
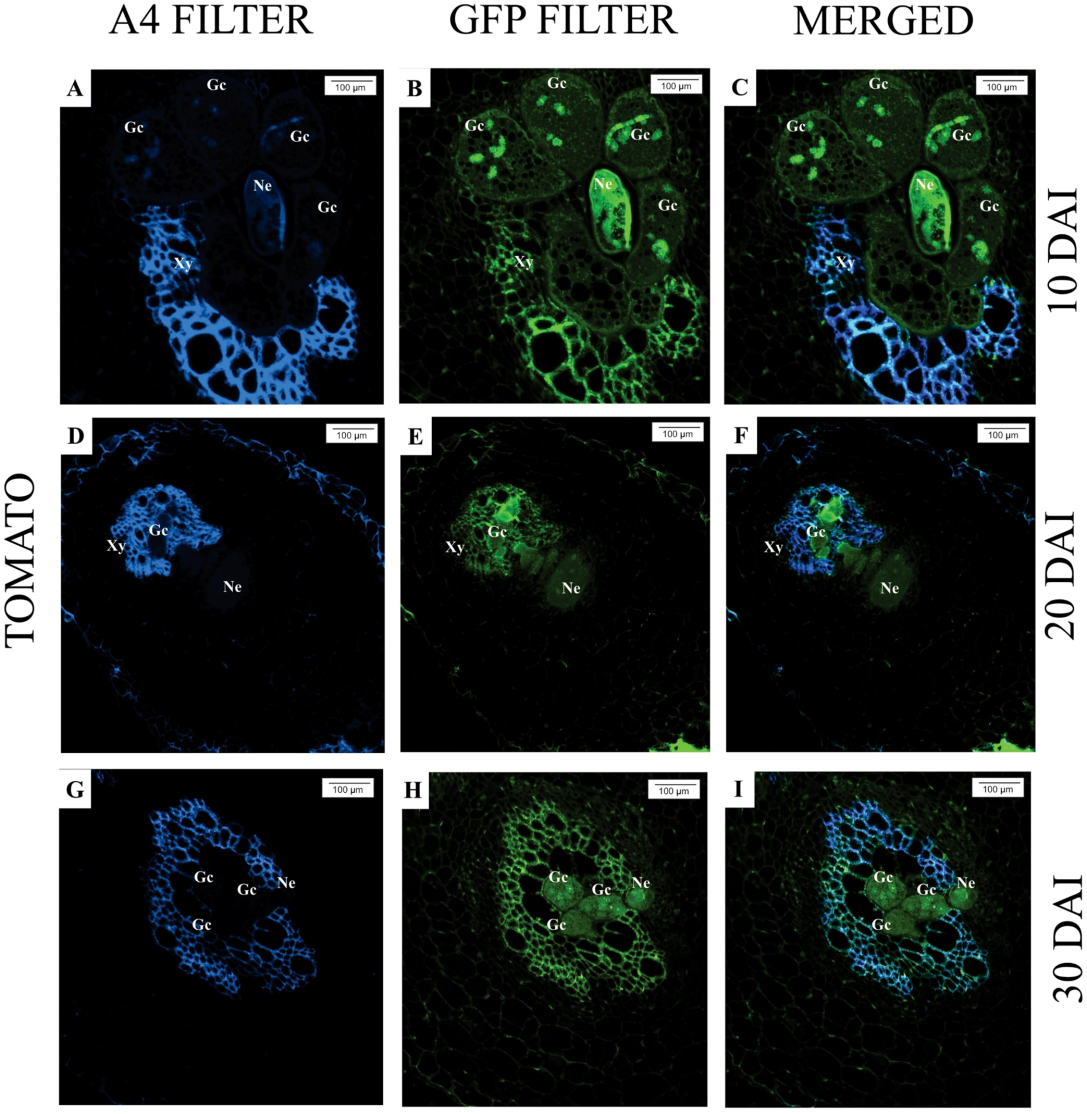
Epifluorescence microscopy analysis of roots of *Macrotyloma axillare* ‘Java’ parasitized by *Meloidogyne javanica* at different days after inoculation (DAI). All images result from the autofluorescence emitted by plant tissues and the nematode (Ne) when excited under different wavelengths. **(A–C)**: Notice the nematode in the intercellular spaces with phenolic compounds (Pc); **(D–F, G–I)**: Female surrounded by cells that accumulated phenolic compounds (Pc) and malformed giant cells (arrow). The endodermis (En) encased the nematode, and adjacent cells exhibited phenolic compounds. Xy, xylem.

Upon analyzing the samples under an epifluorescence microscope, at 10 days after inoculation (DAI), the presence of *M. javanica* was observed in the intercellular spaces of *M. axillare* ‘Java’, although no evident formation of feeding sites (giant cells) was detected, in addition to the presence of phenolic compounds near the infection site (**Figures 3A–C**). At 20 DAI, a female *M. javanica* was observed in the roots of ‘Java’, surrounded by cells accumulating phenolic compounds, along with poorly formed feeding sites (**Figures 3D–F**). At 30 DAI, similar observations were made, with the presence of female *M. javanica* in the roots of *M. axillare* ‘Java’, however, the cells at the feeding site did not present characteristics of giant cells, only cellular disorganization. Furthermore, the endodermis surrounded the nematode infection site, accumulating phenolic compounds (**Figures 3G–I**).

**Figure 4:**
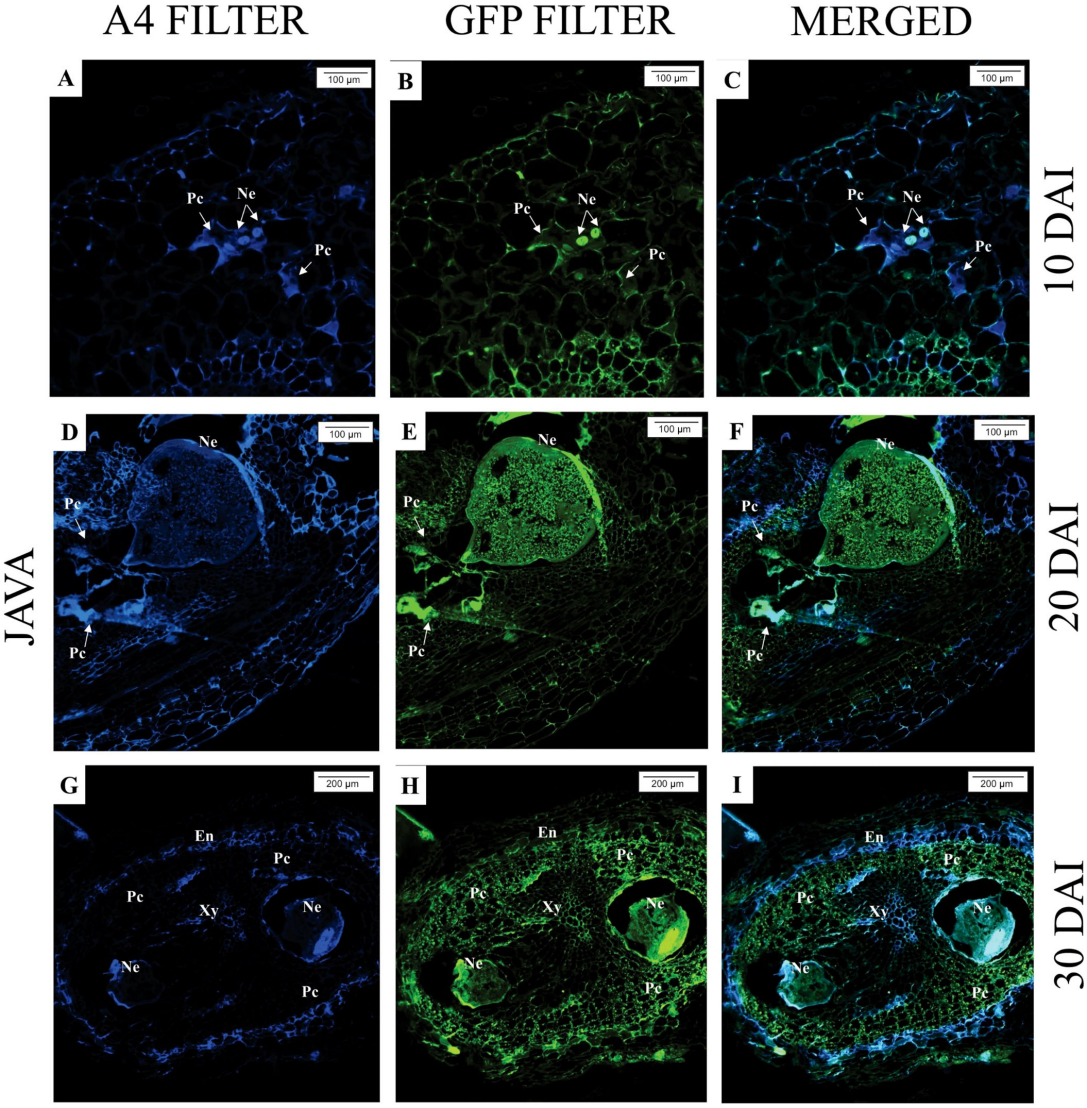
Epifluorescence microscopy analysis of *Solanum lycopersicum* ‘Santa Clara’ roots parasitized by *Meloidogyne javanica* at different days after inoculation (DAI). All images are the result of autofluorescence emitted by plant tissues and the nematode (Ne) when excited under different wavelengths. **(A–C; D–F; G–I)**: At all DAI, induction of giant cells (Gc) by the nematode was observed. Xy, xylem.

In *S. lycopersicum*, for all evaluated times (10, 20, and 30 DAI), the presence of *M. javanica* in the roots can be observed, as well as well-established feeding sites (giant cells), with larger and multinucleated cells, characterizing the successful nematode infection (**Figures 4A–I**).

**Figure 5:**
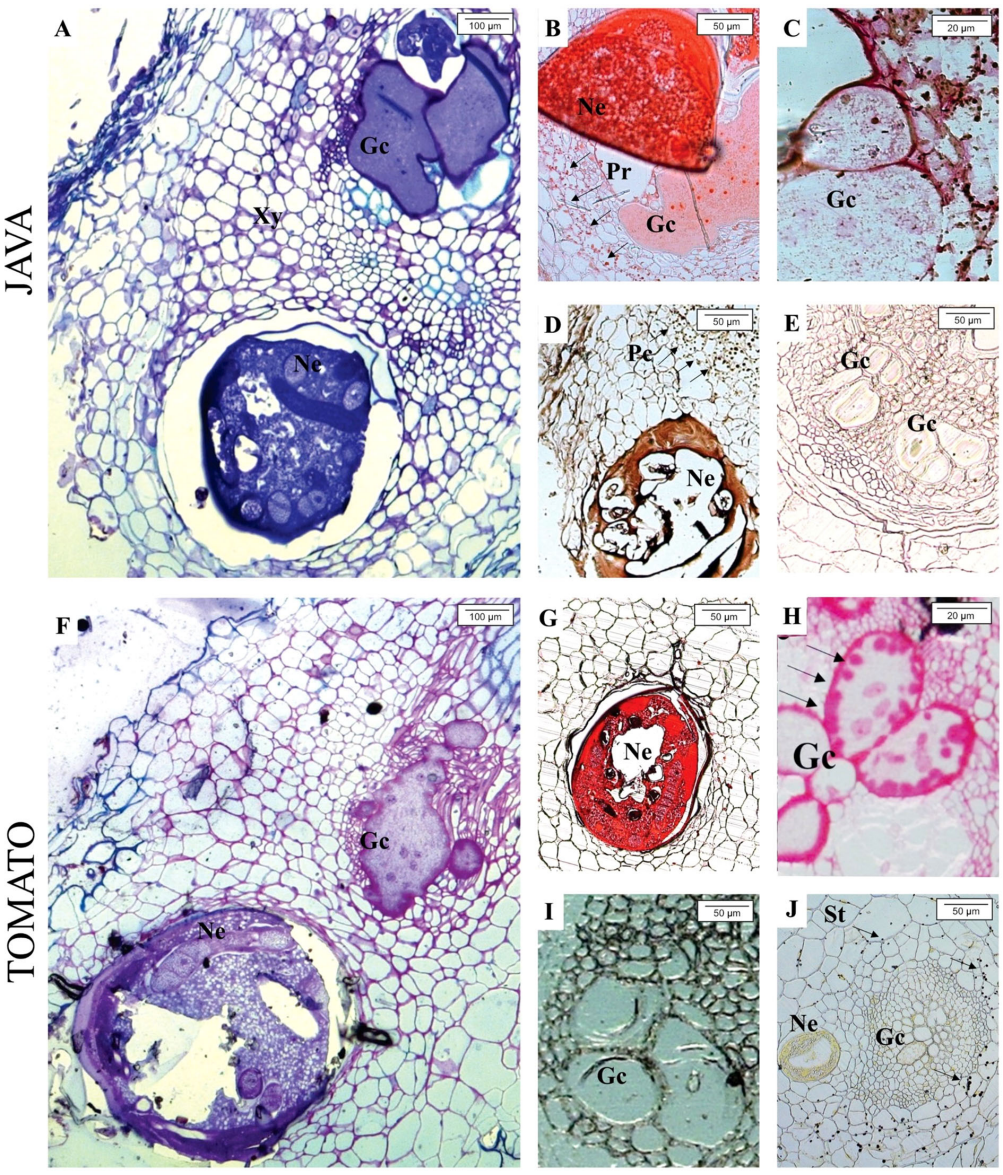
Light microscope analysis of roots of *Macrotyloma axillare* ‘Java’ and *Solanum lycopersicum* ‘Santa Clara’ parasitized by *Meloidogyne javanica* at 30 days after inoculation (DAI). In ‘Java’, the following are observed: **(A)** Nematode (Ne) inducing giant cells (Gc); **(B)** cells around the feeding site with protein accumulation (Pr); **(C)** absence of pectin and starch in the cell wall of giant cells; **(D)** phenolic compounds (Pc) in cells of the feeding site; **(E)** absence of starch in cells of the feeding site. In *S. lycopersicum* ‘Santa Clara’, the following are observed: **(F)** nematode inducing giant cells; **(G)** absence of proteins in cells of the feeding site; **(H)** presence of pectin (Pec) in the cell wall of giant cells; **(I)** absence of phenolic compounds around the giant cells; **(J)** presence of starch (St) in cells of the feeding site. Xy, xylem.

Through toluidine blue staining, at 30 days after inoculation (DAI), the presence of *M. javanica* and giant cells was observed in the roots of *M. axillare* ‘Java’ and *S. lycopersicum* (**Figures 5A, F**). While, when stained with Ponceau Xylidine dye, protein accumulation around the nematode infection site was observed (**Figure 5B**), and in *S. lycopersicum*, protein accumulation was also noted (**Figure 5G**). When stained with Ruthenium Red, feeding sites were observed in both ‘Java’ and *S. lycopersicum* roots, although only in *S. lycopersicum* did the giant cells present pectic invaginations (**Figures 5C, H**). Iron chloride staining revealed phenol accumulation in cells adjacent to the *M. javanica* infection site in ‘Java’, whereas no such compound was present in *S. lycopersicum* (**Figures 5D, I**). Roots of ‘Java’ stained with iodized zinc chloride did not show the presence of starch around the infection site, while in *S. lycopersicum*, starch accumulation was observed in cells adjacent to the nematode infection site (**Figures 5E, J**).

### 3.3 Reactive oxygen species

**Figure 6:**
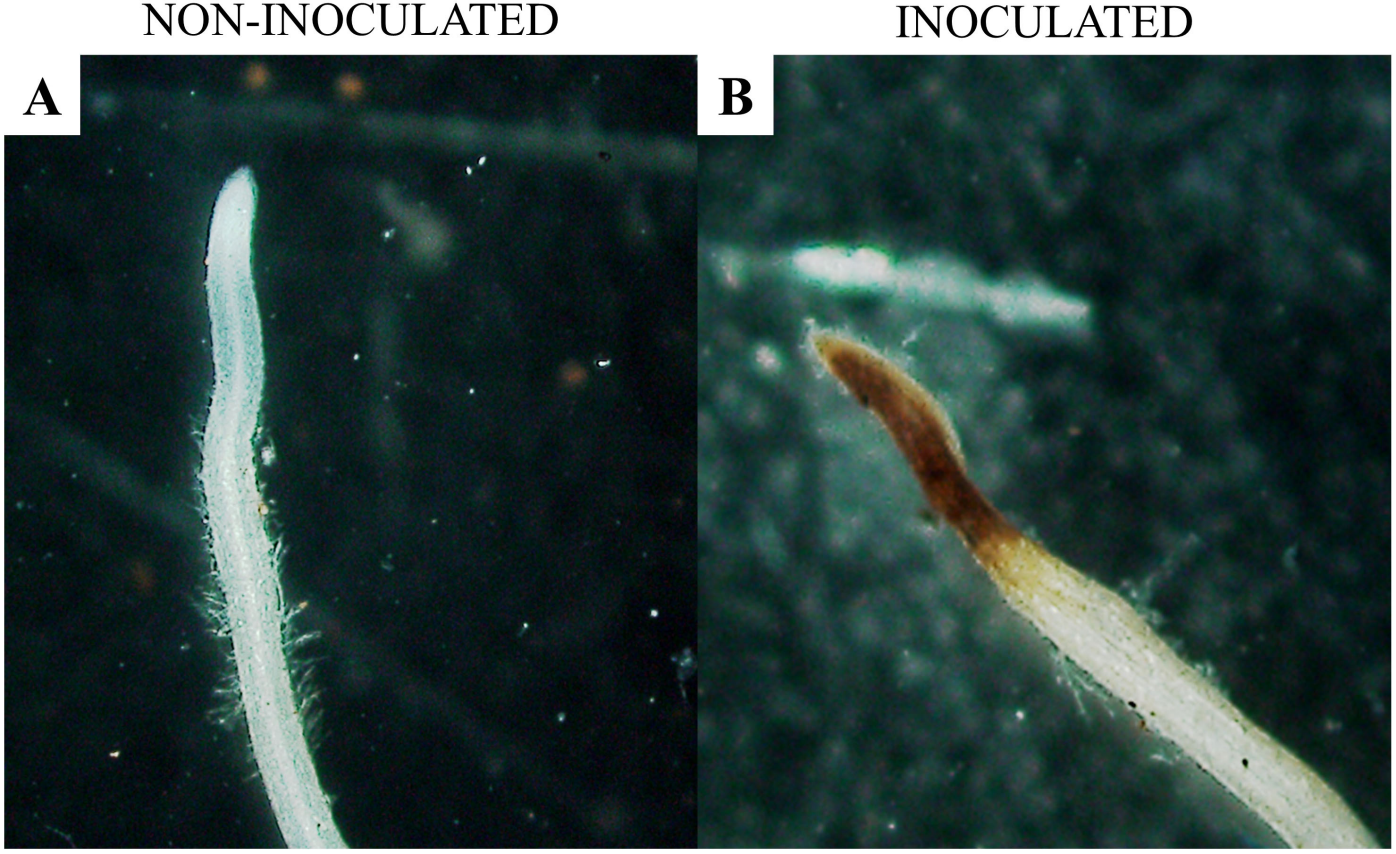
Microscopic analysis of *Macrotyloma axillare* ‘Java’ roots inoculated or not with *Meloidogyne javanica*. **(A)** absence of the reaction with 3,3’-diaminobenzidine (DAB) in the absence of the nematode; **(B)** presence of the reaction with 3,3’-diaminobenzidine (DAB) oxidized by hydrogen peroxide in roots inoculated with the nematode.

**Figure 6A** shows a healthy root segment of Macrotyloma axillare 'Java'. The hypothesis that *M. axillare* ‘Java’ expresses a greater quantity of ROS was accepted by the chi-square test (x²), in which a p-value of 0.004 was found. Out of the 100 root segments evaluated, *M. axillare* ‘Java’, when inoculated with *M. javanica*, showed 42% of samples with a positive reaction to 3,3’-diaminobenzidine (**Figure 6B**), while in *S. lycopersicum* inoculated with the nematode, only 23% showed a reaction to 3,3’-diaminobenzidine.

### 3.4 Scanning electron microscopy

**Figure 7:**
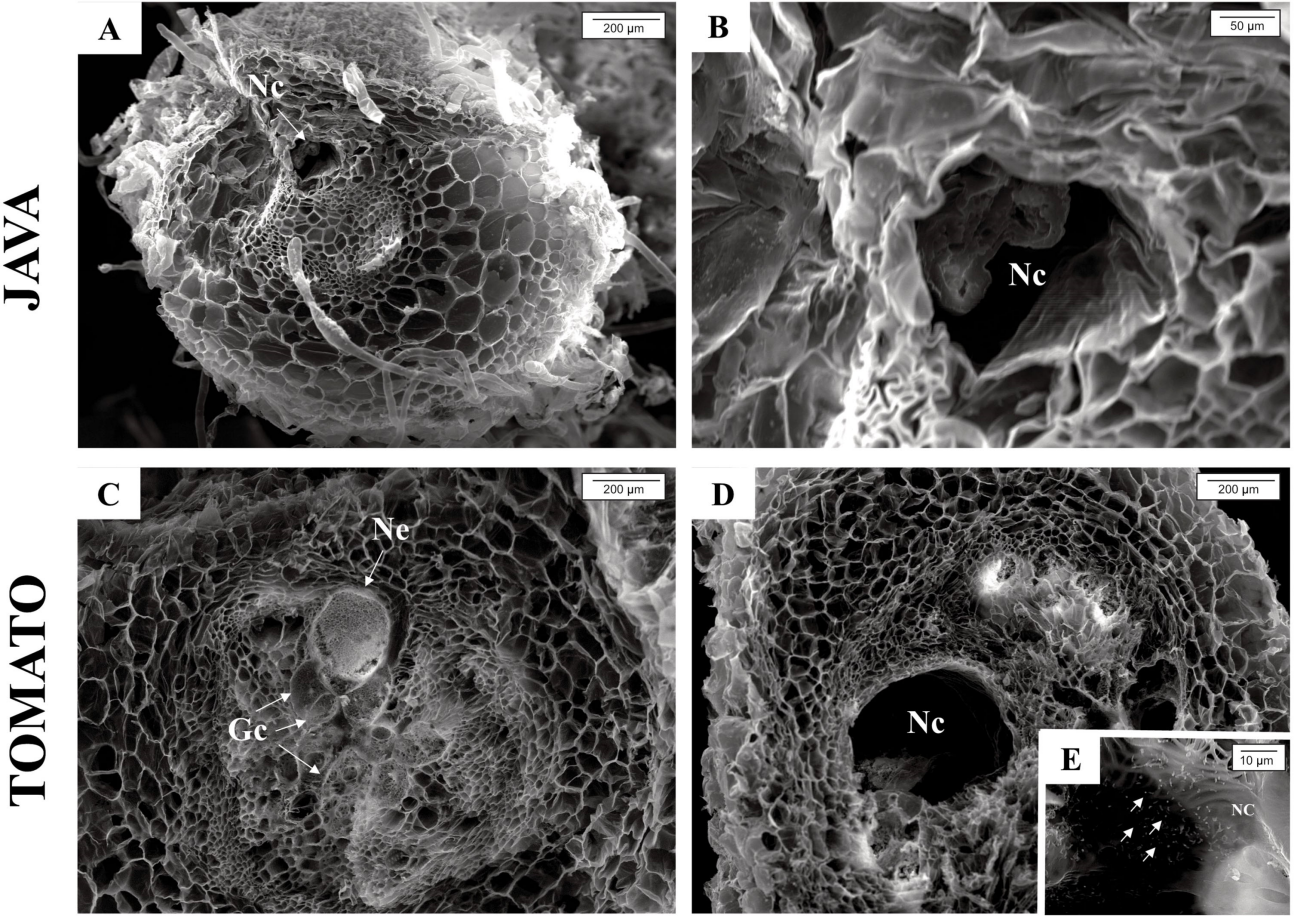
Scanning electron microscopy images showing: *Macrotyloma axillare* ‘Java’ **(A, B)** cavity where the nematode is inserted; *Solanum lycopersicum* ‘Santa Clara’ **(C)** formation of giant cells and presence of the nematode; **(D)** cavity where the nematode is inserted; **(E)** presence of eggs in the nematode’s cavity. Gc, giant cells; Ne, nematode; Nc, Nematode cavity.

Through scanning electron microscopy, it was possible to measure the giant cells in *M. axillare* ‘Java’ and *S. lycopersicum* roots (**Figure 7**). *Macrotyloma axillare* ‘Java’ exhibited giant cells that were, on average, 46% smaller than those observed in *S. lycopersicum*, with values of 2955 μm² and 5572 μm², respectively. These values ​​refer to the averages of 10 giant cells observed in materials from *M. axillare* ‘Java’ and *S. lycopersicum.* Furthermore, in the cavity where the nematode is inserted, the presence of eggs was observed in *S. lycopersicum* roots, indicating nematode reproduction (**Figure 7E**).

### 3.5 Quantification of total proteins

**Figure 8:**
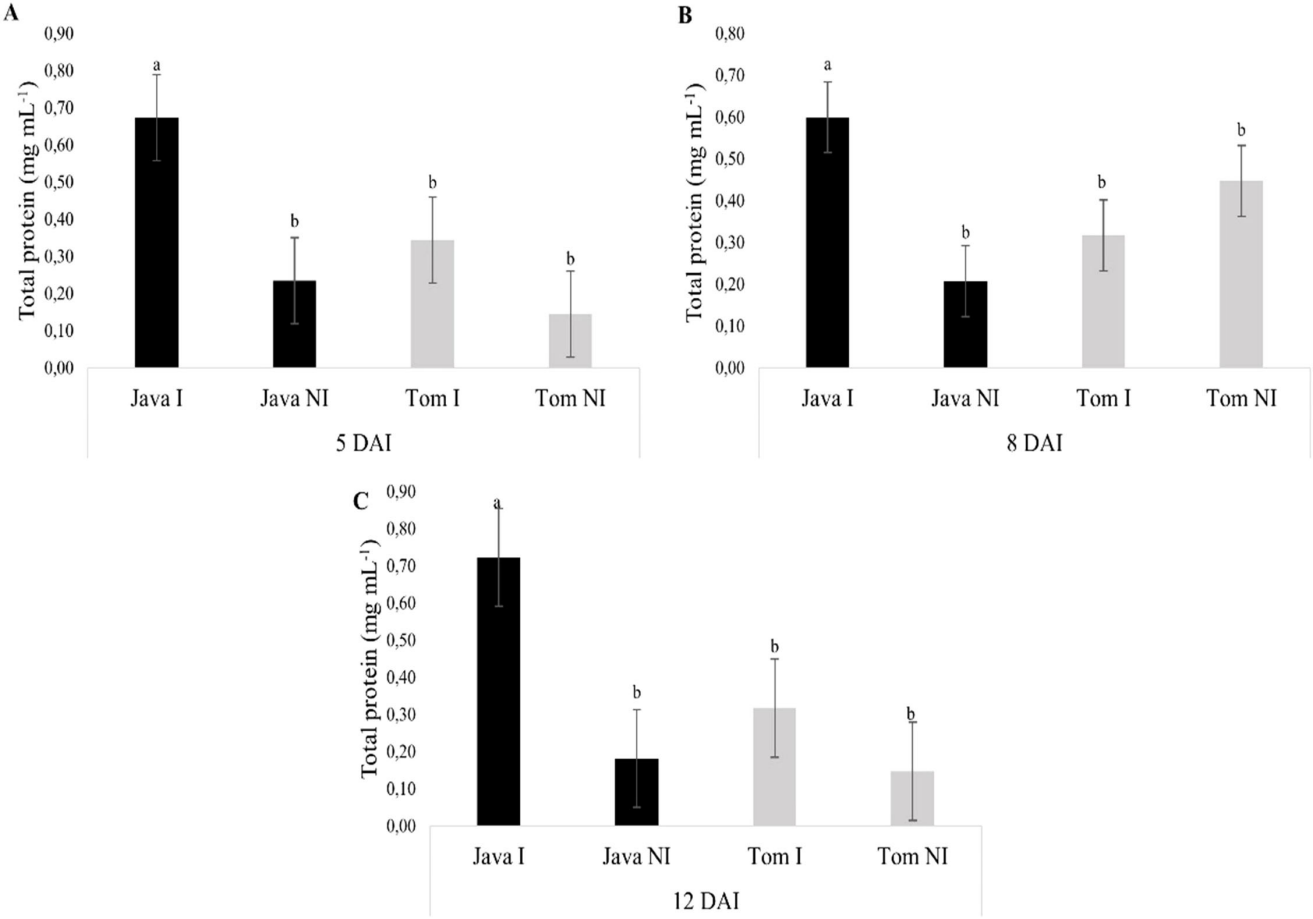
Specific activity of total proteins in roots of *Macrotyloma axillare* 'Java' and *Solanum lycopersicum* 'Santa Clara', inoculated or not with *Meloidogyne javanica*, evaluated at **(A)** 5, **(B)** 8, and **(C)** 12 days after inoculation (DAI). I, Inoculated; NI, Non-inoculated; DAI, Days after inoculation. Bars followed by the same lowercase letter do not differ significantly from each other according to Tukey’s test at 5% probability (p < 0.05).

For all evaluated times (5, 8, and 12 days after inoculation [DAI]), *M. axillare* ‘Java’ inoculated with *M. javanica* exhibited higher total protein concentrations compared to the non-inoculated plant or *S. lycopersicum*, whether inoculated or not (**Figures 8A–C**).

## 4 Discussion

In the present study, *M. axillare* ‘Java’ exhibited resistance to *M. javanica*, as evidenced by a reproduction factor (RF) < 1. Research on this legume is still relatively recent, but previous studies confirm the resistance of *M. axillare* ‘Java’ to *M. javanica* (Miamoto et al., 2016, 2020). Initially, the resistance of *M. axillare* ‘Java’ to the nematode is associated with limited pathogen penetration, and those that manage to penetrate the root system exhibit delayed development (Miamoto et al., 2020). It is also known that the poor development of *M. javanica* within the root system of *M. axillare* ‘Java’ is directly related to the malformed feeding sites, as observed in the present study. Due to the failure to form efficient feeding sites, there is a reduction in the number of adult females and, consequently, a decrease in nematode reproduction (Miamoto et al., 2020). This phenomenon has also been observed in other legumes with a similar mode of action to *M. axillare* ‘Java’, such as *Crotalaria* species, which similarly reduce nematode penetration and negatively impact the formation of feeding sites (Wang et al., 2002).

In *M. axillare* ‘Java’, the formation of giant cells was observed at 30 days after inoculation (DAI), and resistant plants can exhibit multinucleated giant cells with granular cytoplasm and thickened cell walls, similar to those observed in susceptible plants (Regaieg and Horrigue-Raouani, 2012). However, the function of the feeding site formed in resistant plants is not satisfactory, as previously reported in *L. peruvianum* PI126443, a resistant tomato cultivar to *M. incognita*, where giant cells are induced, but in fewer numbers, impairing nematode development (Regaieg and Horrigue-Raouani, 2012). The malformed giant cells observed in *M. axillare* ‘Java’, both in terms of number and size, along with the accumulation of phenolic compounds around the feeding site, are characteristic of a hypersensitive reaction (HR), associated with the nematode’s developmental collapse, as seen in the *Avena sativa* ‘IPR Afrodite’ × *M. incognita* pathosystem (Marini et al., 2016).

The dysfunctionality of the feeding site also reflects the production of antagonistic compounds to the nematode, such as phenolic compounds, which accumulate in cells adjacent to the infection site. The fact that *M. axillare* ‘Java’ produces phenolic compounds in high concentrations has been previously studied; chromatographic analysis of plant extract revealed an abundance of phenolic acids, such as p-hydroxybenzoic and coumaric acids (Miamoto et al., 2022b). These compounds have demonstrated nematicidal effects (Aoudia et al., 2012) and are commonly found near the nematode infection site, reducing the pathogen’s feeding capacity, thus acting as a plant resistance factor (Oliveira et al., 2019).

Through epifluorescence microscopy analysis, it was observed that the endodermis of *M. axillare*’Java’ roots surrounded the nematode infection site, forming a protective barrier. Additionally, the presence of suberin in the endodermis of *M. axillare* ‘Java’ was noted, even in the absence of the nematode. Endodermal cell walls with suberin lamellae generally hinder nutrient absorption, but this can be an efficient solution for protecting cells against pathogen attacks. To establish effective colonization, many pathogens manipulate the host cell function and immune response by injecting effector molecules into the cell (or otherwise transporting them across the plasma membrane). Suberin lamellae may be an effective means of rendering endodermal cells refractory to manipulation by bacteria or fungi (Geldner, 2013).

The presence of reactive oxygen species (ROS) following inoculation with the nematode was also evident in *M. axillare* ‘Java’ roots. ROS are known as “oxidative bursts” which aim to make the cell inhospitable to the pathogen, resulting in reactions such as the hypersensitive response (HR) (Mohammadi et al., 2021). In this regard, the presence of polyphenol oxidase and peroxidase also explains the ROS observed in the present study, as these enzymes act as antioxidants, playing a role in decomposing oxidative cells that, in high concentrations, may cause damage to plants (Zhang and Sun, 2021).

In the present study, it was observed that roots of *M. axillare* ‘Java’ inoculated with *M. javanica* exhibited higher concentrations of total proteins, corroborating the findings from the histochemical tests. The accumulation of resistance proteins in *M. axillare* ‘Java’ had been previously identified, with higher concentrations of polyphenol oxidase, peroxidase, β-1,3-glucanases, and phenylalanine ammonia-lyase observed when the plant was inoculated with the nematodes (Miamoto et al., 2022a), highlighting post-infection (induced) resistance.

Further studies are needed to elucidate the interaction between *M. axillare* ‘Java’ and *M. javanica*. Future research involving gene expression analyses at the transcriptomic and proteomic levels may provide a deeper understanding of the molecular mechanisms underlying this interaction.

## 5 Conclusion

It was concluded that *M. axillare* ‘Java’ presents both constitutive and induced defense characteristics, through the production of phenolic compounds and likely defense proteins. This inhibits the full development of *M. javanica* in its roots, thereby preventing its reproduction.

## Data Availability

The raw data supporting the conclusions of this article will be made available by the authors, without undue reservation.
